# Development and validation of a nomogram for predicting overall survival in patients with sinonasal mucosal melanoma

**DOI:** 10.1186/s12885-024-11888-5

**Published:** 2024-02-07

**Authors:** Zhenzhen Zhu, Weiqing Wang, Yang Zha, Xiaowei Wang, Surita Aodeng, Lei Wang, Yuzhuo Liu, Wei Lv

**Affiliations:** Department of Otolaryngology-Head and Neck Surgery, Peking Union Medical College Hospital, Chinese Academy of Medical Sciences, Peking Union Medical College, No.1, Shuaifuyuan, Wangfujing, Dongcheng District, 100730 Beijing, China

**Keywords:** Sinonasal mucosal melanoma, Prognosis, Nomogram, Survival analysis, SEER database

## Abstract

**Background:**

Sinonasal mucosal melanoma (SNMM) is a relatively rare malignant tumour with a poor prognosis. This study was designed to identify prognostic factors and establish a nomogram model to predict the overall survival (OS) of patients with SNMM.

**Methods:**

A total of 459 patients with SNMM were selected from the Surveillance, Epidemiology, and End Results (SEER) database as the training cohort. Univariate and multivariate Cox regression analyses were used to screen for independent factors associated with patient prognosis and develop the nomogram model. In addition, external validation was performed to evaluate the effectiveness of the nomogram with a cohort of 34 patients with SNMM from Peking Union Medical College Hospital.

**Results:**

The median OS in the cohort from the SEER database was 28 months. The 1-year, 3-year and 5-year OS rates were 69.8%, 40.4%, and 30.0%, respectively. Multivariate Cox regression analysis indicated that age, T stage, N stage, surgery and radiotherapy were independent variables associated with OS. The areas under the receiver operating characteristic curves (AUCs) of the nomograms for predicting 1-, 3- and 5-year OS were 0.78, 0.71 and 0.71, respectively, in the training cohort. In the validation cohort, the area under the curve (AUC) of the nomogram for predicting 1-, 3- and 5-year OS were 0.90, 0.75 and 0.78, respectively. Patients were classified into low- and high-risk groups based on the total score of the nomogram. Patients in the low-risk group had a significantly better survival prognosis than patients in the high-risk group in both the training cohort (*P* < 0.0001) and the validation cohort (*P* = 0.0016).

**Conclusion:**

We established and validated a novel nomogram model to predict the OS of SNMM patients stratified by age, T stage, N stage, surgery and radiotherapy. This predictive tool is of potential importance in the realms of patient counselling and clinical decision-making.

**Supplementary Information:**

The online version contains supplementary material available at 10.1186/s12885-024-11888-5.

## Introduction

Sinonasal mucosal melanoma (SNMM) is a rare malignant entity characterized by a poor prognosis; it constitutes 0.7-1% of all melanomas and 4–8% of all malignancies in the nasal cavity and paranasal sinus [1]. However, the pathophysiology of SNMM has not been elucidated. Compared with cutaneous melanoma, mucosal melanoma (MM) often manifests with advanced stages and aggressive behaviour. The absence of specific symptoms may contribute to the delayed diagnosis of SNMM. Primary treatment for SNMM revolves around surgery and encompasses both endoscopic and external approaches [1]. Although adjuvant radiotherapy, chemotherapy, immunotherapy and targeted therapy have been adopted in the multimodal treatment of SNMM, the recurrence rate is still high, and the 5-year overall survival rate is less than 30% [2].

The eighth edition of the American Joint Committee on Cancer (AJCC) staging manual introduced a TNM staging system for MM of the head and neck, which is distinct from the staging system for sinonasal carcinoma [2]. However, no prognostic stage grouping for MM has been proposed, possibly due to the lack of robust data supporting the relationship between AJCC stages and survival prognosis. A nomogram is a simple graphical presentation of a prediction model that generates probabilities of a specific clinical endpoint [3]. The predictive ability of the nomogram was superior to that of the TNM staging system for many cancers [4, 5]. Consequently, nomograms have found widespread utility in prognostic predictions and individualized treatment planning across diverse cancer types. Several factors associated with survival outcome have been identified in small retrospective case series [6, 7] and population-based studies [8, 9]. The Surveillance, Epidemiology, and End Results (SEER) database is an authoritative source of cancer data in the United States that could provide adequate cases to construct a prognostic prediction model for rare tumours. In this study, we developed a new nomogram for predicting the survival of patients with SNMM based on data from the SEER database. External validation was also performed to evaluate the effectiveness of the nomogram using the SNMM cohort from our centre.

## Methods

### Study patients

**Fig. 1:**
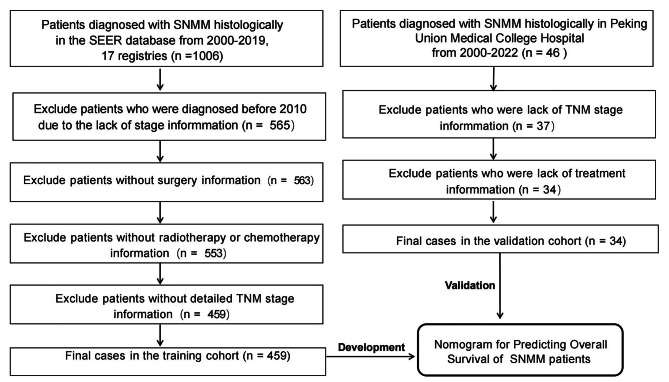
The flow chart of patient selection and study design

SEER Stat software (SEER*Stat, v8.4.0.1) was used to extract clinical data from 2000 to 2019 from the SEER database (SEER Research Plus Data from 17 Registries). The screening and exclusion criteria were as follows: (1) the primary sites of the tumour were the nasal cavity and paranasal sinus, coded as C30.0, C31.0-C31.3, C31.8, and C31.9 according to the International Classification of Disease for Oncology, Third Edition (ICD-O-3) topography; (2) the tumour was pathologically confirmed as mucosal melanoma, coded as 8720–8772 according to ICD-O-3; and (3) patients without complete clinicopathological, treatment and survival data were excluded. A total of 459 patients with SNMM were ultimately recruited in the training cohort from the SEER database. In addition, patients diagnosed and treated for SNMM between 2000 and 2022 at the Department of Otolaryngology Head and Neck Surgery, Peking Union Medical College Hospital between 2000 and 2022 were selected. Patients without complete demographic, clinicopathological and survival information were excluded. Consequently, 34 patients were included in the validation cohort from our institution. In this study, all patients from the SEER database and our centre were staged according to the seventh edition of the AJCC TNM Cancer Staging Manual. The flow chart of patient selection and study design is depicted in Fig. 1. Due to the study design, this study had an institutional review board exemption (I-23ZM0066) and was conducted following the principles outlined by the Declaration of Helsinki.

### Data extraction and end point

Variables, including age, sex, race, pathological diagnosis, primary site, AJCC stage, T stage, N stage, M stage, surgery, radiotherapy, chemotherapy, survival months and vital status, were extracted for analysis. The primary endpoint of the study was overall survival (OS). Survival time was calculated from the date of diagnosis to the date of the last follow-up or until the date of death due to any cause.

### Statistical analysis

Statistical analysis was performed using R software (version 4.2.0, http://www.r-project.org). The overall survival curves were plotted through the Kaplan‒Meier method and analysed with the log-rank test. To identify the prognostic factors associated with OS, variables satisfying *P* < 0.1 in the univariate Cox regression analysis were included in the multivariate Cox regression analysis. Variables with a *P* value less than 0.05 in the multivariate analysis were regarded as independent prognostic factors. We constructed a nomogram model for predicting 1-, 3- and 5-year OS with these independent risk factors. A calibration curve comparing the mean predicted survival rate with the actual survival rate was used to verify the discriminatory ability of the nomogram. Decision curve analysis (DCA) was performed by calculating the net benefits for a range of threshold probabilities and was used to estimate the clinical usefulness of the nomogram [10]. The predictive ability of the nomogram model in both the training cohort and the validation cohort was determined by receiver operating characteristic (ROC) curve and area under the curve (AUC) analyses. Based on the nomogram, we calculated the total score for each patient. The optimal cutoff value of the total nomogram score was determined through X-tile software (version 3.6.1). The net reclassification index (NRI) was calculated to evaluate the reclassification ability of the nomogram compared to the 7th edition of the AJCC-TNM staging system.

## Results

### Clinical characteristics

A total of 459 patients were selected from the SEER database as the training cohort. Among them, 28.5% were younger than 65 years, 41.0% were between 65 and 79 years, and 30.5% were older than 80 years. The sex distribution was 44.2% male and 55.8% female; 85% (85.0%) were identified as White, and 15.0% represented other racial backgrounds. The primary site was the nasal cavity in 79.7% of the patients in the training cohort, while the remaining 20.3% of the lesions originated in the paranasal sinus. Additionally, 34 patients from our institution composed the validation cohort, with 64.7%, 32.4%, and 2.9% falling into the respective age groups above; 38.2% were male, and 61.8% were female. The detailed demographic and clinical characteristics are presented in Table 1.

**Table 1:** Demographic and clinical characteristics of patients with SNMM in the training and validation cohort

Variable	Training Cohort from SEER database		Validation Cohort from China		*P* value
	N	%	N	%	
**Age(years)**	459	100.0	34	100.0	< 0.001
< 65	131	28.5	22	64.7	
65–79	188	41.0	11	32.4	
≥ 80	140	30.5	1	2.9	
**Gender**					0.592
Male	203	44.2	13	38.2	
Female	256	55.8	21	61.8	
**Race**					< 0.001
White	390	85.0	0	0.0	
Others	69	15.0	34	100.0	
**Primary site**					0.049
Nasal cavity	366	79.7	22	64.7	
Paranasal sinus	93	20.3	12	35.3	
**T Stage**					0.244
T3	256	55.8	14	41.2	
T4a	148	32.2	14	41.2	
T4b	55	12.0	6	17.6	
**N Stage**					0.595
N0	403	87.8	29	85.3	
N1	56	12.2	5	14.7	
**M Stage**					
M0	409	89.1	26	76.5	0.047
M1	50	10.9	8	23.5	
**TNM Stage**					0.111
III	225	49.0	14	41.2	
IVA	137	29.9	7	20.6	
IVB	47	10.2	5	14.7	
IVC	50	10.9	8	23.5	
**Surgery**					
No	76	16.6	5	14.7	1.000
Yes	383	83.4	29	85.3	
**Radiotherapy**					
No	164	35.7	17	50.0	0.101
Yes	295	64.3	17	50.0	
**Chemotherapy**					< 0.001
No	422	91.9	10	29.4	
Yes	37	8.1	24	70.6	
Death event					0.469
No	182	39.7	11	32.4	
Yes	277	60.3	23	67.6	

### Survival analysis and screening of prognostic factors

In the training cohort, the median follow-up time was 18 months (interquartile range: 8 to 39 months), and the median OS was 28 months (95% confidence interval (CI): 24–32 months). The 1-year, 3-year and 5-year OS rates were 69.8% (95% CI, 65.6–74.3%), 40.4% (95% CI, 35.7–45.7%), and 30.0% (95% CI, 25.4–35.5%), respectively. In the validation cohort, the median follow-up time was 30.5 months (interquartile range: 12 to 65 months), with a median OS of 39 months (95% CI, 21–132 months). The 1-year, 3-year and 5-year OS rates were 70.6% (95% CI, 56.8–87.7%), 53.6% (95% CI, 38.7–74.3%) and 42.1% (95% CI, 27.6–64.5%), respectively.

**Fig. 2:**
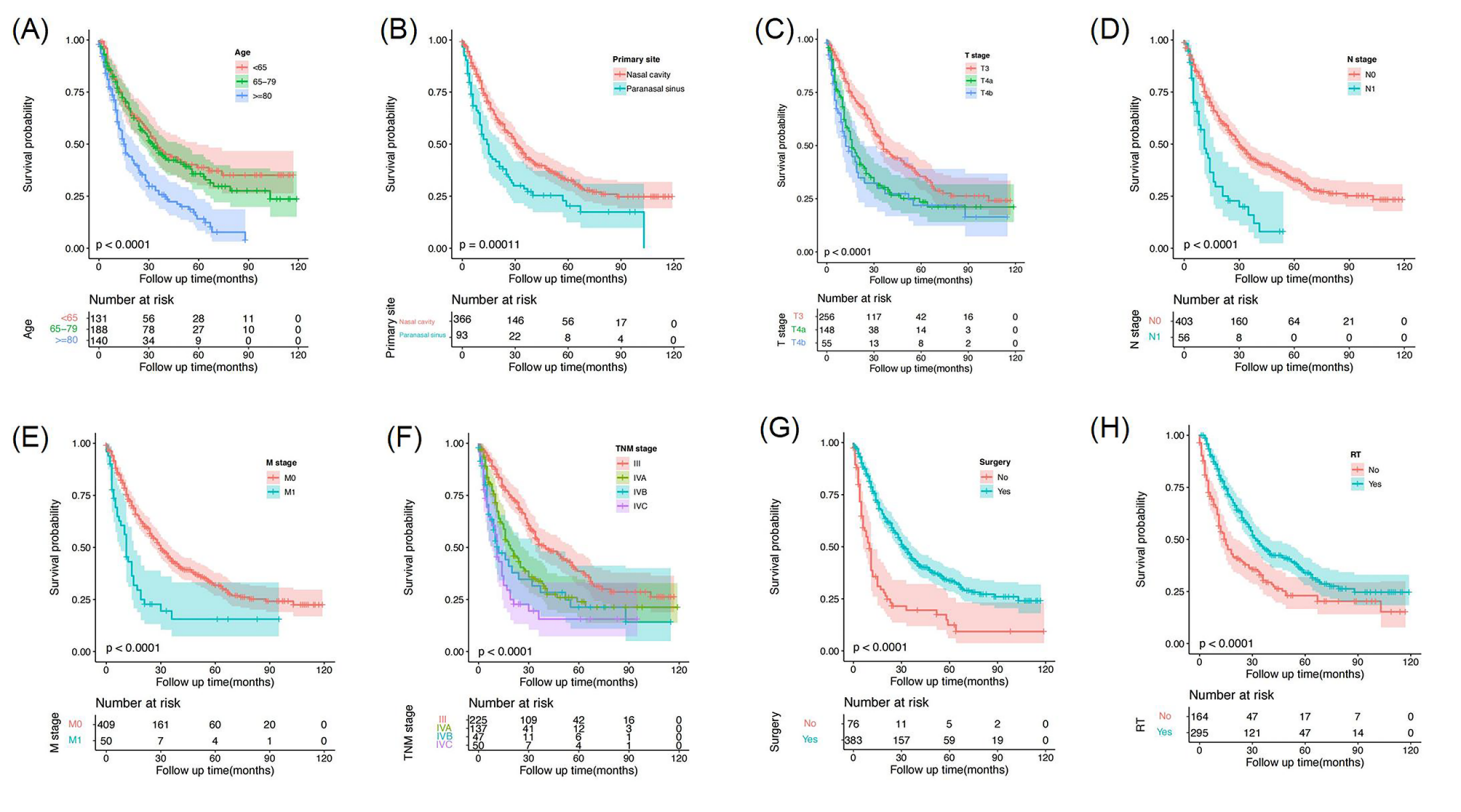
Kaplan–Meier curves of overall survival in patients with sinonasal mucosal melanoma by age (**A**), primary site (**B**), T stage (**C**), N stage (**D**), M stage (**E**), TNM stage (**F**), surgery (**G**), and radiotherapy (**H**). RT, radiotherapy

To identify prognostic variables, univariate Cox regression analysis of the OS of SNMM patients from the SEER database was conducted. The following variables were related to OS: age, primary site, T stage, N stage, M stage, TNM stage, surgery and radiotherapy. OS curves based on the Kaplan‒Meier method and log-rank test were used to visualize the different survival outcomes stratified by different parameters (Fig. 2). Significant differences in OS were detected between subgroups according to age, primary site, T stage, N stage, M stage, TNM stage, surgery and radiotherapy. These variables, except for TNM stage, were included in the multivariate Cox regression analysis. The TNM stage is a combination of the AJCC T, N, and M stages instead of an independent variable. Furthermore, multivariate Cox regression analysis revealed that age, T stage, N stage, surgery and radiotherapy were significant independent risk factors for OS (Table 2, *P* < 0.05).

**Table 2:** Univariate and multivariate cox regression analysis in the training cohort

Variable	Univariate analysis		Multivariate analysis	
	HR (95% CI)	*P* value	HR (95% CI)	*P* value
**Age (years)**				
< 65	Reference		Reference	
65–79	1.14(0.84–1.54)	0.413	1.16(0.85–1.57)	0.358
≥ 80	2.09(1.54–2.84)	**< 0.001**	1.99(1.46–2.71)	**< 0.001**
**Gender**				
Male	Reference		-	
Female	0.98(0.77–1.24)	0.845	-	
**Race**				
White	Reference		-	
Others	0.87(0.61–1.24)	0.437	-	
**Primary site**				
Nasal cavity	Reference		Reference	
Paranasal sinus	1.71(1.30–2.25)	**< 0.001**	1.27(0.92–1.75)	0.148
**T Stage**				
T3	Reference		Reference	
T4a	1.68(1.30–2.18)	**< 0.001**	1.37(1.02–1.85)	**0.038**
T4b	1.85(1.29–2.66)	**< 0.001**	1.81(1.24–2.65)	**0.002**
**N Stage**				
N0	Reference		Reference	
N1	2.44(1.74–3.42)	**< 0.001**	2.17(1.52–3.10)	**< 0.001**
**M Stage**				
M0	Reference		Reference	
M1	2.25(1.59–3.17)	**< 0.001**	1.27(0.85–1.89)	0.248
**TNM Stage**				
III	Reference			
IVA	1.70(1.29–2.24)	**< 0.001**		
IVB	2.12(1.42–3.14)	**< 0.001**		
IVC	2.90(2.01–4.20)	**< 0.001**		
**Surgery**				
No	Reference		Reference	
Yes	0.39(0.30–0.53)	**< 0.001**	0.56(0.41–0.77)	**< 0.001**
**Radiotherapy**				
No	Reference		Reference	
Yes	0.58(0.46–0.74)	**< 0.001**	0.62(0.48–0.79)	**< 0.001**
**Chemotherapy**				
No	Reference		-	
Yes	1.21(0.82–1.78)	0.349	-	

HR, hazard ratio; CI, confidence interval;

### Development and validation of the nomogram model

**Fig. 3:**
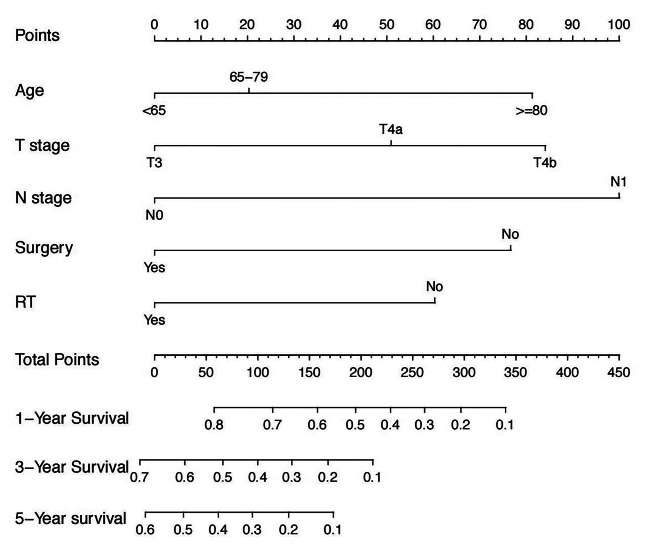
Nomogram model predicting 1-, 3- and 5-year OS for patients with SNMM. RT, radiotherapy. OS, overall survival

**Fig. 4:**
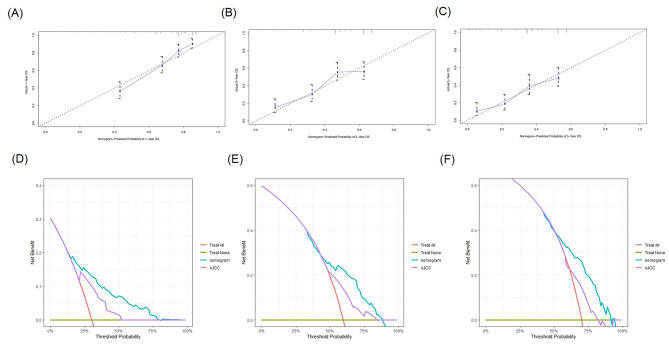
Calibration curves of the nomogram for the prediction of the 1-year (**A**), 3-year (**B**), and 5-year (**C**) overall survival probability in the training cohort. Decision Curve Analysis (DCA) of the nomogram and the 7th AJCC TNM stage for 1-year (**D**), 3-year (**E**) and 5-year (**F**) OS in training set. The turquoise line represents the nomogram and the purple line represents AJCC TNM stage. AJCC, American Joint Committee on Cancer. OS, overall survival

A nomogram incorporating these five significant independent prognostic factors identified through multivariate Cox regression analysis was established (Fig. 3), and the detailed score for each variable is shown in Supplementary Table 1. The total score of these factors was used to predict each patient’s 1-year, 3-year, and 5-year survival probabilities. The C-index was 0.700 (95% CI, 0.669–0.731). The time-dependent C-index values for the nomogram were greater than those of the 7th edition of the AJCC TNM staging system (Supplementary Fig. 1). The calibration curves demonstrated that the predicted survival results of the nomogram corresponded well to the actual survival rates (Fig. 4A-C). In addition, the DCA plots showed that the nomogram presented a greater clinical net benefit than the AJCC TNM staging system (Fig. 4D-F). The AUC of the nomogram for predicting 1-, 3- and 5-year OS was 0.78, 0.71 and 0.71, respectively, in the training cohort (Fig. 6A).

**Fig. 5:**
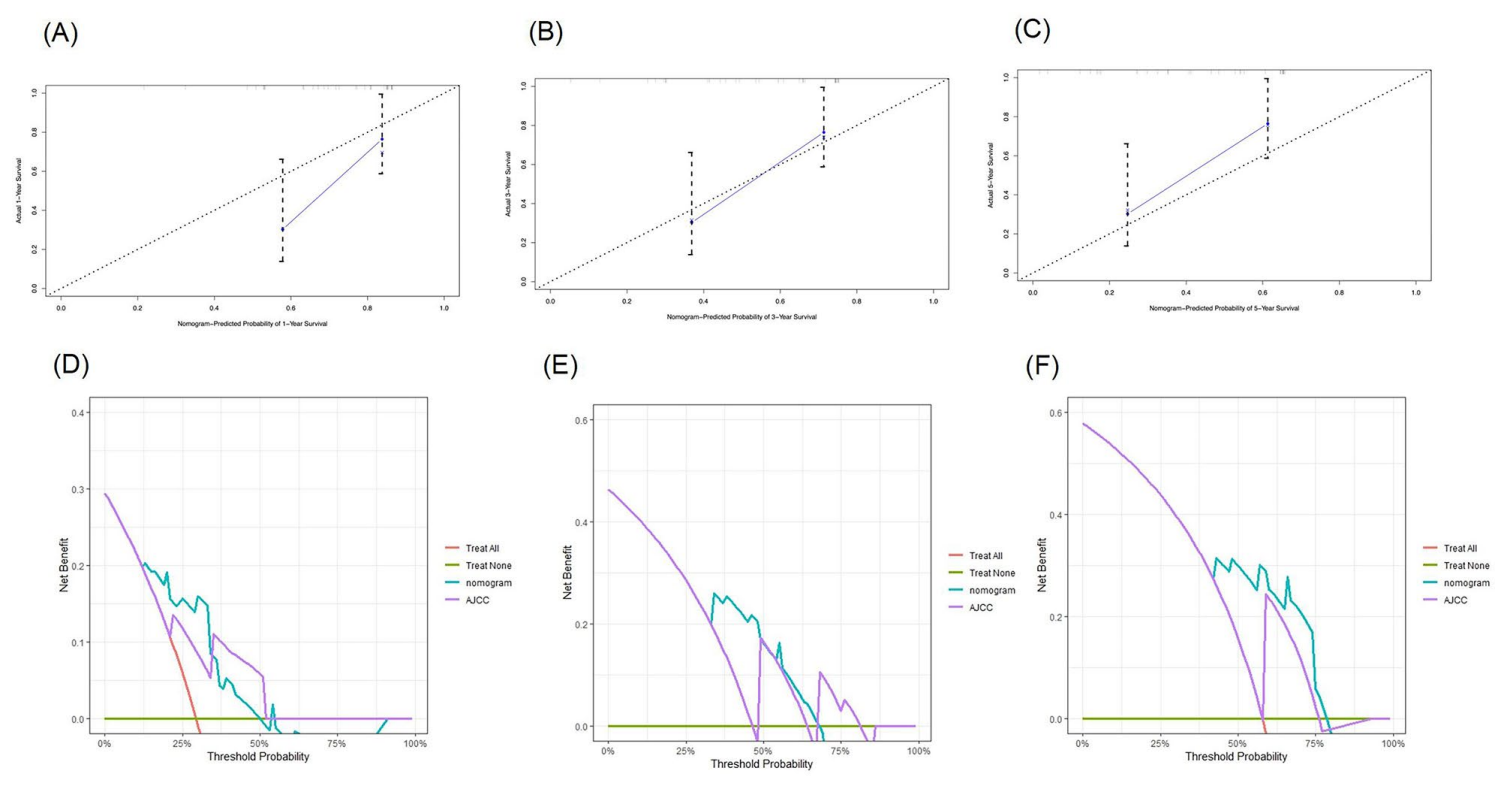
Calibration curves of the nomogram for the prediction of the 1-year (**A**), 3-year (**B**), and 5-year (**C**) overall survival probability in the external validation cohort. Decision Curve Analysis (DCA) of the nomogram and the 7th AJCC TNM stage for 1-year (**D**), 3-year (**E**) and 5-year (**F**) OS in validation set. The turquoise line represents the nomogram and the blue line represents AJCC TNM stage. AJCC, American Joint Committee on Cancer. OS, overall survival

**Fig. 6:**
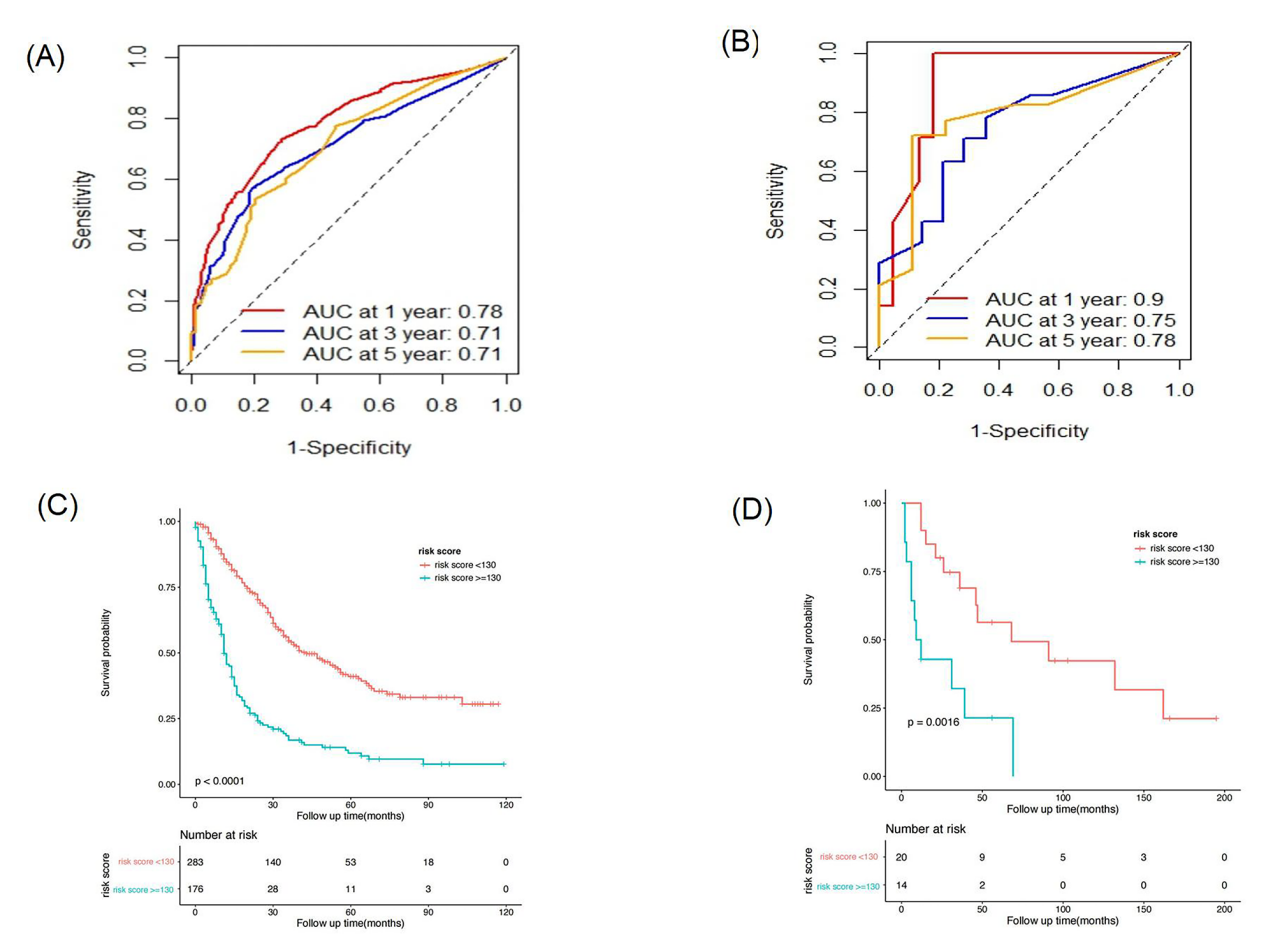
The receiver operating characteristic (ROC) curves of the nomogram predicting 1-, 3- and 5-year OS in the training cohort (**A**) and the validation cohort (**B**). Kaplan-Meier curves of OS for the low-risk (risk score < 130) and high-risk (risk score ≥ 130) groups in the training cohort (**A**) and the validation cohort (**B**). OS, overall survival. AUC, area under the curve

In the validation cohort, the C-index was 0.719 (95% CI, 0.611–0.827). The calibration curves of the nomogram for the validation cohort are shown in Fig. 5A-C. The DCA plots comparing the clinical net benefit of the nomogram and the AJCC TNM staging system are shown in Fig. 5D-F. The AUC of the nomogram for predicting 1-, 3- and 5-year OS were 0.90, 0.75 and 0.78, respectively (Fig. 6B).

The NRI values showed that the newly developed model had a significantly greater proportion of correctly reclassified patients than did the AJCC staging system. In the SEER cohort, the NRI values for 1-, 3-, and 5-year OS were 0.243 (95% CI 0.065–0.419), 0.165 (95% CI 0.021–0.390) and 0.154 (95% CI 0.005–0.295), respectively. In the validation cohort, the NRI values for 1-, 3-, and 5-year OS were 0.358 (95% CI -0.385–0.952), -0.017 (95% CI -0.439–0.892) and 0.465 (95% CI-0.230–0.967), respectively.

We calculated the total points based on the nomogram model, and the optimal cutoff value was determined to be 130 by X-tile software. Therefore, patients with a total score (also called the risk score) less than 130 points were classified into the low-risk group, and those whose total score was equal to or greater than 130 points were classified into the high-risk group. K‒M curves demonstrated that the patients in the low-risk subgroup had a significantly better survival prognosis than did those in the high-risk subgroup in both the training cohort (*P* < 0.0001; Fig. 6C) and the validation cohort (*P* = 0.0016; Fig. 6D).

For SNMM patients without distant metastasis according to the SEER database (*n* = 409), age, primary site, T stage, N stage, surgery and radiotherapy were identified as significant independent risk factors associated with OS through multivariate Cox regression analysis (Supplementary Table 2). We also constructed a prognostic nomogram for patients with the six significant factors mentioned above (Supplementary Fig. 2).

## Discussion

Although its incidence is increasing, SNMM is one of the most common malignant head and neck tumours [11]. Treatment of SNMM is highly challenging due to the high rate of local recurrence and distant metastasis. Therefore, it is highly important to construct an intuitive prognostic model to predict survival and facilitate individualized treatment strategies. In previous studies, scholars have developed prognostic nomograms for head and neck mucosal melanoma based on the variables age, location, T stage, N stage, and surgery [12, 13]. This study was designed to establish a prognostic nomogram model specifically for the survival outcome of patients with SNMM based on data from the SEER database. We identified five independent variables using univariate and multivariate Cox regression analyses and established a novel nomogram to predict the prognosis of SNMM. Advanced age at diagnosis, high T stage and N stage were associated with worse prognosis, while surgery and radiotherapy were associated with improved survival. Moreover, the nomogram was validated using an external cohort from our centre, which yielded satisfactory results.

Patients with SNMM are usually diagnosed in their fifth to eighth decade of life, and the median age is 65–70 years [1, 2]. Advanced age was shown to be associated with decreased survival in patients with SNMM [8, 14, 15]. The sex distribution of SNMM is similar [1], and male sex was regarded as a negative prognostic predictor of overall survival in patients with SNMM in a retrospective study from a single institution [6]. However, no significant correlation between sex and survival outcome was detected in our study or in others [7, 9, 15]. For SNMM, the most common primary site is the nasal cavity rather than the paranasal sinus. As reported in previous studies [8, 16–18], the prognosis of patients with mucosal melanoma originating from the paranasal sinuses is poorer than that of patients with melanoma arising from the nasal cavity. On the one hand, lesions in the nasal cavity are easier to detect than those in the paranasal sinuses. On the other hand, patients with tumours arising from the nasal cavity are more likely to go to a doctor due to early symptoms such as epistaxis and nasal congestion. Therefore, melanomas arising from paranasal sinus are more likely to be diagnosed at late stage, and the patients lose the chance of radical surgery. Specifically, in this study, the primary site was an independent prognostic factor for patients without systemic tumour burden but not for those with metastatic disease. One explanation is that the prognosis of patients with metastatic disease is poor regardless of the primary site.

Surgical resection remains the cornerstone of SNMM treatment. In recent years, with the advancements in endoscopic techniques, endoscopic approaches have been used in the resection of sinonasal malignancies. Notably, survival outcomes are similar between patients who have undergone endoscopic resection of SNMM and patients who have undergone open surgery [15, 16, 19]. The impact of radiotherapy on the survival of patients with SNMM is controversial. A previous meta-analysis revealed that adjuvant radiotherapy could prolong the survival of SNMM patients compared with surgery alone [20]. However, radiotherapy was not associated with overall survival in a series of 1874 patients with SNMM from the National Cancer Database [15]. Our study revealed that the survival rate of patients who have undergone surgery and radiotherapy is better than that of patients who have not.

Recently, targeted therapies, including inhibitors of *c-KIT*, *NRAS/MEK* or *BRAF*, and immunotherapies, including anti-CTLA-4 and anti-PD-1/PD-L1 antibodies, have revolutionized the treatment of cutaneous melanoma. Although mucosal melanoma patients were excluded from the majority of clinical trials [21], patients with mucosal melanoma could also benefit from targeted and immunologic therapy [22, 23]. For SNMM patients with distant metastases, immunotherapy was associated with improved survival [15]. Combination therapies with nivolumab and ipilimumab demonstrated superior efficacy compared to treatment with individual drugs [24]. Zebary et al. showed that *KIT* and *BRAF* mutations were rare in SNMM, while *NRAS* mutations were relatively frequent [25]. Similar results were achieved by Amit et al. and Chraybi et al. [26, 27], who reported that these mutations could be used for direct targeted therapy. Targeted therapy has provided promising results in the treatment of SNMM [23, 28]. Cao et al. recently reported that an SNMM patient with a *ROS1* fusion achieved complete remission after 8 months of treatment with crizotinib [29]. However, more robust evidence from multicentre prospective studies is needed for targeted and immunologic therapy for SNMM.

This study has several limitations. First, the clinical variables available in the SEER database were limited. For example, tumour size, immunotherapy, targeted therapy, surgical approach and margin status were not available from the SEER database and were not included in the analytical process. In previous studies, a positive surgical margin was identified as one of the negative prognostic factors for SNMM [6, 26]. Second, pathological characteristics and mutation data were not included in this study, although several pathological characteristics, such as the mitotic index and the expression of Ki-67, PD-1 and IDO-1, have been found to be associated with the survival outcome of SNMM [30, 31]. Third, the median follow-up time was 18 months (IQR, 8 to 39 months) in the training cohort; thus, the nomogram exhibited weaker predictive power for 3- and 5-year OS. In addition, the sample size of the validation cohort from our single centre was small, and the verification results might change when the nomogram is validated in a larger external cohort. Large-scale prospective multicentre cohorts will be needed in the future to validate and optimize the nomogram.

## Conclusion

In summary, we found that age, T stage, N stage, surgery and radiotherapy were independent risk factors for OS in SNMM patients. In response to these discerned factors, we successfully devised and rigorously validated a novel nomogram model. This predictive tool is of potential importance in the realms of patient counselling and clinical decision-making, offering a systematic and data-driven approach to prognostication for individuals grappling with SNMM.

### Electronic supplementary material

Below is the link to the electronic supplementary material.

Supplementary Material 1

## Data Availability

The datasets used and analyzed during the current study are available in SEER database (http://seer.cancer.gov) and from the corresponding author on reasonable request.
