# Supplementary material for: Development and validation of a nomogram for predicting overall survival in patients with sinonasal mucosal melanoma
Source: BMC Cancer. 2024 Feb 7;24:184. doi: 10.1186/s12885-024-11888-5 (PMC10851497; doi:10.1186/s12885-024-11888-5)
Supplement: Supplementary file 1 — Supplementary Material 1 [file 12885_2024_11888_MOESM1_ESM.docx]

Supplementary Material

**Development and Validation of a Nomogram for Predicting Overall Survival in Patients with Sinonasal Mucosal Melanoma**

**Zhenzhen Zhu^1^, Weiqing Wang^1^, Yang Zha^1^, Xiaowei Wang^1^, Aodeng Surita^1^, Lei Wang^1^, Yuzhuo Liu^1^, and Wei Lv^1^***

**Affiliation:** ^1^Department of Otolaryngology-Head and Neck Surgery, Peking Union Medical College Hospital, Chinese Academy of Medical Sciences, Peking Union Medical College, Beijing, China

***Corresponding author：**Wei Lv, MD

Department of Otolaryngology-Head and Neck Surgery, Peking Union Medical College Hospital, Chinese Academy of Medical Sciences, Peking Union Medical College, No.1, Shuaifuyuan, Wangfujing, Dongcheng District, Beijing 100730, China.

E-mail: [lili20020615@sina.com](mailto:lili20020615@sina.com)

**Supplementary Table 1. Detailed scores for each variable in the nomogram**

| **Variable** | **Score** |
| --- | --- |
| **Age (years)** |  |
| <65 | 0 |
| 65-79 | 20.3 |
| ≥80 | 81.3 |
| **T Stage** |  |
| T3 | 0 |
| T4a | 50.9 |
| T4b | 84.1 |
| **N Stage** |  |
| N0 | 0 |
| N1 | 100 |
| **Surgery** |  |
| No | 76.6 |
| Yes | 0 |
| **Radiotherapy** |  |
| No | 60.3 |
| Yes | 0 |

**Supplementary Table 2 Multivariate cox regression analysis in patients without distant metastasis**

| **Variable** | **Multivariate analysis** | |
| --- | --- | --- |
|  | **HR (95% CI)** | ***P* value** |
| **Age (years)** |  |  |
| <65 | Reference |  |
| 65-79 | 1.39(0.98-1.96) | 0.061 |
| ≥80 | 2.31(1.64-3.26) | **<0.001** |
| **Primary site** |  |  |
| Nasal cavity | Reference |  |
| Paranasal sinus | 1.77(1.27-2.46) | **<0.001** |
| **T Stage** |  |  |
| T3 | Reference |  |
| T4a | 1.41(1.03-1.93) | **0.034** |
| T4b | 2.25(1.50-3.37) | **<0.001** |
| **N Stage** |  |  |
| N0 | Reference |  |
| N1 | 2.56(1.70-3.84) | **<0.001** |
| **Surgery** |  |  |
| No | Reference |  |
| Yes | 0.66(0.46-0.95) | **0.024** |
| **Radiotherapy** |  |  |
| No | Reference |  |
| Yes | 0.61(0.47-0.80) | **<0.001** |

HR, hazard ratio; CI, confidence interval;

**Supplementary Figure 1**


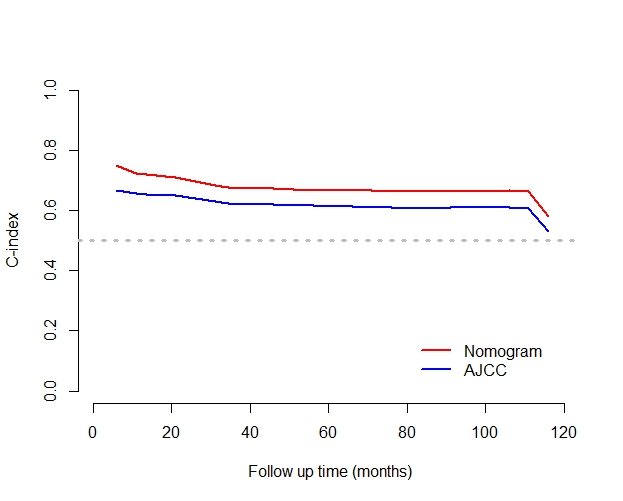


**Supplementary Figure 1.** The time-dependent C-index values for the nomogram and AJCC TNM staging system

**Supplementary Figure 2**


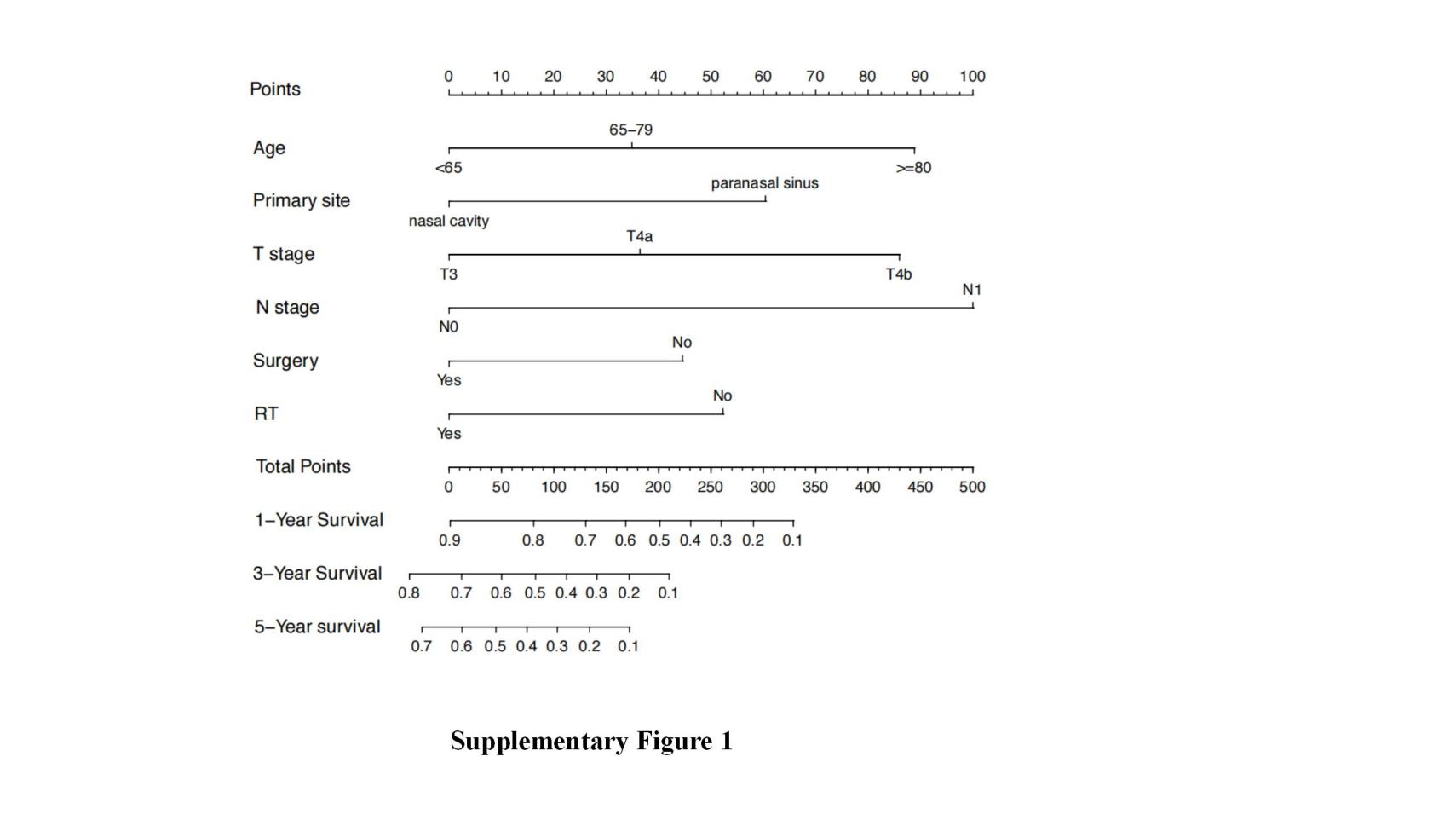


**Supplementary Figure 2.** Nomogram model predicting 1-, 3- and 5-year OS for SNMM patients without distant metastasis. RT, radiotherapy. OS, overall survival.
